# Developing a spatial-statistical model and map of historical malaria prevalence in Botswana using a staged variable selection procedure

**DOI:** 10.1186/1476-072X-6-44

**Published:** 2007-09-24

**Authors:** Marlies H Craig, Brian L Sharp, Musawenkosi LH Mabaso, Immo Kleinschmidt

**Affiliations:** 1 Malaria Research Programme, Medical Research Council, 491 Ridge Road, Overport, Durban, 4091, South Africa; 2 Swiss Tropical Institute, 57 Socinstrasse, Basel, BS 4002, Switzerland; 3 London School of Hygiene and Tropical Medicine, Keppel Street, London WC1E 7HT, UK

## Abstract

**Background:**

Several malaria risk maps have been developed in recent years, many from the prevalence of infection data collated by the MARA (Mapping Malaria Risk in Africa) project, and using various environmental data sets as predictors. Variable selection is a major obstacle due to analytical problems caused by over-fitting, confounding and non-independence in the data. Testing and comparing every combination of explanatory variables in a Bayesian spatial framework remains unfeasible for most researchers. The aim of this study was to develop a malaria risk map using a systematic and practicable variable selection process for spatial analysis and mapping of historical malaria risk in Botswana.

**Results:**

Of 50 potential explanatory variables from eight environmental data themes, 42 were significantly associated with malaria prevalence in univariate logistic regression and were ranked by the Akaike Information Criterion. Those correlated with higher-ranking relatives of the same environmental theme, were temporarily excluded. The remaining 14 candidates were ranked by selection frequency after running automated step-wise selection procedures on 1000 bootstrap samples drawn from the data. A non-spatial multiple-variable model was developed through step-wise inclusion in order of selection frequency. Previously excluded variables were then re-evaluated for inclusion, using further step-wise bootstrap procedures, resulting in the exclusion of another variable. Finally a Bayesian geo-statistical model using Markov Chain Monte Carlo simulation was fitted to the data, resulting in a final model of three predictor variables, namely summer rainfall, mean annual temperature and altitude. Each was independently and significantly associated with malaria prevalence after allowing for spatial correlation. This model was used to predict malaria prevalence at unobserved locations, producing a smooth risk map for the whole country.

**Conclusion:**

We have produced a highly plausible and parsimonious model of historical malaria risk for Botswana from point-referenced data from a 1961/2 prevalence survey of malaria infection in 1–14 year old children. After starting with a list of 50 potential variables we ended with three highly plausible predictors, by applying a systematic and repeatable staged variable selection procedure that included a spatial analysis, which has application for other environmentally determined infectious diseases. All this was accomplished using general-purpose statistical software.

## Background

Recent years have seen widespread application of geographic information systems and spatial statistical methods in modelling and mapping the distribution of vector borne diseases, including malaria. In sub-Saharan Africa the Mapping Malaria Risk in Africa (MARA) project has been working towards a malaria risk atlas for rational and targeted control of the disease [1]. To this end historical and current survey data have been collated of the prevalence of infection with human *Plasmodium *parasites.

A number of malaria risk maps, at country and regional level, have been produced by analysing geo-referenced prevalence data against environmental data to predict prevalence at localities where it was not recorded [2-6]. Different analytical approaches of varying sophistication have been explored. Multiple variable logistic regression analysis, commonly used to assess the odds of infection against potential risk factors, has been employed, and the spatial dependence in the response data has been modelled most successfully using Bayesian spatial modelling. One outstanding issue, which can greatly affect the predictions, remains the variable selection procedure, particularly when there are a large number of potential risk factors.

In regression analysis and predictive/prognostic statistics, model validity is an important aspect [7], both the internal validity, or accuracy, i.e. the model explains the observed data well, and external validity, or generalizability, i.e. the model predicts new data well. In this context we furthermore aim for parsimony (model contains a few strong predictors that are easily interpretable) and plausibility, both of the co-variates (association with the disease are etiologically explainable) and of the predictions (believable in view of what is generally known). Taking account of the spatial correlation structure in the data is important for "geographic transportability", i.e. when predicting malaria prevalence to unobserved locations [8].

Selecting a few predictors for spatial modelling from among a large number of potential candidates is a major challenge and can easily become arbitrary. Ideally every possible combination of variables would be tested and compared in a Bayesian spatial framework. However, this would be extremely computing-intensive and unfeasible, if not impossible, for most users. The most practical route is to reduce the list of potential explanatory variables using non-spatial selection methods, before moving to a spatial context.

Neither manual nor automated stepwise selection procedures are advised, because of frequent over-fitting, and because of the resulting "phantom degrees of freedom" [9] pg 416: testing and rejecting many variables increases the probability of finding a significant predictor by chance, but since this sifting remains undeclared, standard errors in the final model are underestimated. Babyak [9], citing Harrell [10] and others, recommend shorter lists of candidate predictor variables, which are not strongly correlated, as well as bootstrapping, as a form of simulation. Austin and Tu [11], working on heart attack data, developed their model by running repeated step-wise selection procedures on bootstrap samples of their data, to identify the most consistent predictors.

The aim of this study was to develop a map of historical malaria risk for Botswana by analysing malaria prevalence data against a number of environmental variables from different data themes, using a systematic and repeatable staged process of variable elimination, including the stepwise bootstrap method described by Austin and Tu [11]. The resulting small subset of variables, each independently associated with the response, but possibly spurious because the condition of spatial independence was not satisfied, was tested in a Bayesian geo-statistical model. We used the spatial model derived from the observed locations, to predict prevalence of malaria infection in children 1–14 years old at unobserved map locations across the whole country.

## Methods

### Study area

**Figure 1:**
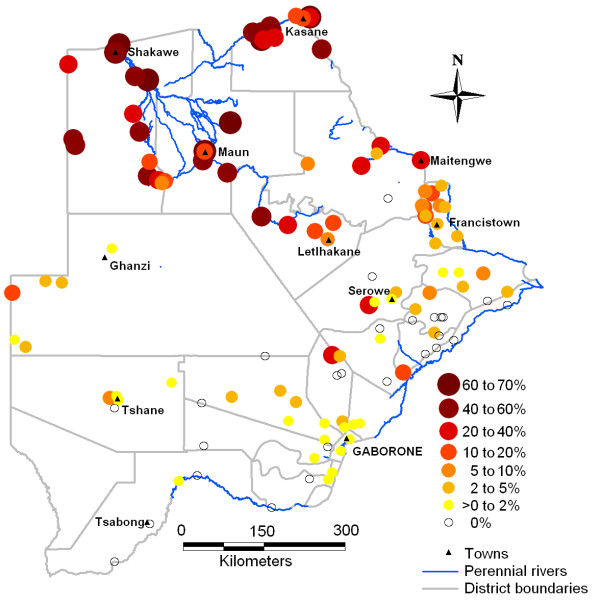
**Malaria prevalence data**. Malaria prevalence of infection in 1 to 14 year old children, in Botswana, during the 1961/62 national survey.

Botswana is semi-arid to arid with few permanent water bodies. The country is flat, mostly between 900 and 1200 m altitude. The rainy season is from November to March. Vegetation ranges from desert scrub-land in the South-West, where annual rainfall is <300 mm, through grassland, to wooded savannah in the North, which receives >500 mm rain annually. Mean annual temperatures are between 18 and 23°C. Botswana today has a total population of about 1.6 million; population density over two thirds of the country being <1 per square km [12]. The population according to the 1971 census was 630379 with an approximate 3.1% annual increase [13] which if extrapolated back in time translates to around 470000 in 1961/62. In 1975 80% of the population lived in the eastern part of the country,

Malaria risk is highest in the tropical North (figure 1). Indoor residual spraying was introduced in 1946 on a limited scale. Coverage was gradually improved culminating in a comprehensive vector control program in the 1980's [14], but even by 1953 indoor residual spraying for mosquito control was a "regular feature" in risk areas, apparently mainly in towns, along rivers and apparently excluding rural areas "remote from regular medical supervision", but with good results [15]. Larval control was also implemented when mosquito breeding was detected. Malaria prevalence decreased markedly after 1944, again between 1961/62 and 1974, and further thereafter [14]. By 1960 no prevalence above 70% was measured, suggesting meso-to hypo-endemic conditions. Further South, transmission is hypo-endemic and epidemic, and over large areas entirely absent. Incidence, like the climate, is strongly seasonal, peaking around March/April [16]. The gradient in malaria broadly follows the environmental gradients described before.

### Malaria data

**Figure 2:**
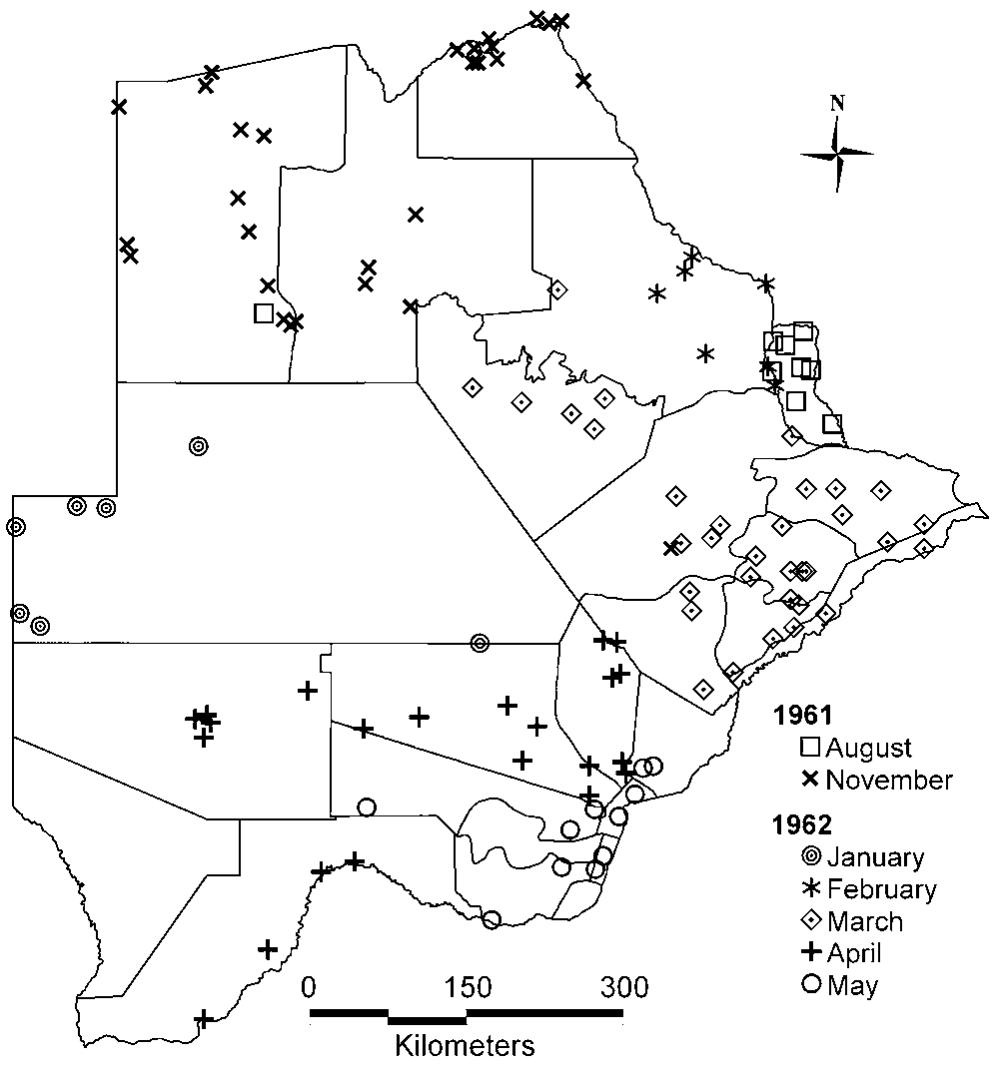
Month of survey during the 1961/62 national malaria survey.

Archived malaria prevalence data were collated within the MARA project, as described by Omumbo *et al *[17]. In Botswana geographical coordinates could be obtained for 613 out of a total of 1063 age-specific prevalence surveys. Of these, 20 did not report sample sizes and were excluded. Here we used only the 1961/62 national survey (figure 1) to develop a historical malaria risk map. For the 1–14 year age group, 122 prevalence results were available, for 118 unique locations across the country, progressively from August 1961 to May 1962. Surveys in different regions were carried out during different months (figure 2). The total number examined was 17149; the mean sample size was 141 per survey (range 2–831). The design effect was calculated in Stata [18].

### Environmental data

Forty-nine variables representing different summaries and transformations of the eight environmental data themes (see table 1), were included in the study: elevation [19], surface water [20], land cover [21], long-term monthly mean rainfall, temperature [22], vapour pressure [23], and normalized difference vegetation index (NDVI) at 8 km [24] and 1 km [25] resolution.

**Table 1:** Results of uni-variate analysis from Stage 1. Odds Ratios (AIC in parentheses) from univariate logistic regression analysis of 50 different environmental variables from 7 themes, against malaria prevalence. P-values were non-significant (n.s.), <0.05(*), <0.01(**) or <0.0005 (***), n = 122. The equation was logit(prevalence) = coefficient × co-variate + constant. NDVI = normalized difference vegetation index.

	**Environmental data theme**					
**Variable**	**Rain-fall (mm)**	**Temperature (°C)**	**Vapor pressure (hPa)**	**NDVI, 8 km resolution **§	**NDVI, 1 km resolution **§	**Other**
Annual mean (total for rainfall)	1.0085 (27.6)**	4.22 (13.6)***	1.094 (12.8)***	1.091 (28.9)*	1.07 (31.3) n.s.	
Annual maximum (highest monthly value)	1.045 (20.8)***	3.034 (23.3)***	1.067 (11.7)***	1.090 (25.7)***	10.4 (32.2) n.s.	
Annual minimum (lowest monthly value)		3.29 (13.9)***	1.11 (17.1)***	1.1048 (29.8)*	1.06 (32.7) n.s.	
Annual range (highest minus lowest month)		0.52 (27.1)**	1.12 (15.8)***	1.14 (30.8)*	1.03 (32.7) n.s.	
Standard deviation (Appendix)	1.03 (21.9)***	0.54 (25.0)***	0.54 (14.7)**	1.073 (26.6)***	1.03 (32.8) n.s.	
Proportional standard deviation (Appendix)‡	61.8 (13.0)***	-214 (17.3)***	0.004 (33.4) n.s.	0.1 (26.8)***	43.3 (32.9) n.s.	
Summer mean (total for rainfall) Dec–Mar	1.012 (22.9)***	2.59 (27.1)***	1.065 (11.6)***	1.078 (28.9)*		
Winter mean (total for rainfall) Apr–Oct	0.88 (14.8)***	3.22 (12.0)***	1.11 (16.0)***	1.097 (28.6)**		
Concentration (see Appendix)	1.39 (13.3)***					
Number of months >80 mm (>60 & >40 mm n.s.)	1.81 (26.6)**					
Number of months >16°C		2.72 (18.9)***				
Number of months >165 (other cut-offs were n.s.)				1.13 (31.5) n.s.		
Total in months with more than 80 mm	1.0059 (24.0)***					
Total degree months above 16°C		1.050 (15.7)***				
Effective temperature (Appendix)		21.8 (12.6)***				
Mean daily minimum of coldest month		2.29 (21.4)***				
Elevation						0.997 (29.7)**
Log distance to perennial water (m)						0.56 (21.6)***
Log distance to perennial/non-perennial water (m)						0.72 (30.5)**
Land cover (binary; moist *versus *dry areas)						4.76 (25.5)***
Month of survey (binary; peak season April/May *versus *rest of year)						8.67 (29.4)***

‡ The co-efficients, not the Odds Ratios, are shown, as the unit is a fraction, and the Odds Ratio near zero (= exp(co-efficient)).

§ Radiance units for NDVI (fractions from 0 to 1) are translated to a byte-compatible scale from 1 to 256.

Themes with monthly values (rainfall, temperature, NDVI and vapor pressure) were plotted against logit-transformed malaria prevalence, logit(p). Based on observed temporal patterns in the scatter plots, months were aggregated for "summer" (December to March) and "winter" (April to October). Different annual summary indices were also calculated for each theme. Calculations of some of the variables are shown in the appendix.

Distance from water bodies was calculated by projecting maps of perennial and non-perennial water bodies onto a 200 × 200 m grid and calculating for each grid cell the euclidian distance to the nearest water body. Values were transformed by adding a value of 100 m to each pixel and deriving the natural logarithm.

For land cover, the thirteen United States Geological Survey land cover classes occurring in Botswana were re-grouped into two categories, broadly corresponding to drier and moister land cover types. Most data points were found in "grassland" and "savannah" with only isolated surveys in the other land cover types. Prevalence was generally higher in "savannah" than in "grassland" areas. Other obviously drier and lower risk land cover types ("barren or sparsely vegetated", "shrub land", "urban or built-up") were therefore included with "grassland" in a "low risk" category, while other clearly moister classes ("herbaceous wetland", "water bodies", "evergreen broadleaf forest") were included with the higher-risk "savannah" category. Other minor land cover types were included in the category alongside which they mostly commonly occurred ("grassland/crop land mosaic" was mainly found scattered among "grassland"; "dryland crop land and pasture" and "mixed" among "savannah").

Values were extracted from the data grids for each geographical location where a malaria survey result was available.

### Variable selection and model development

**Figure 3:**
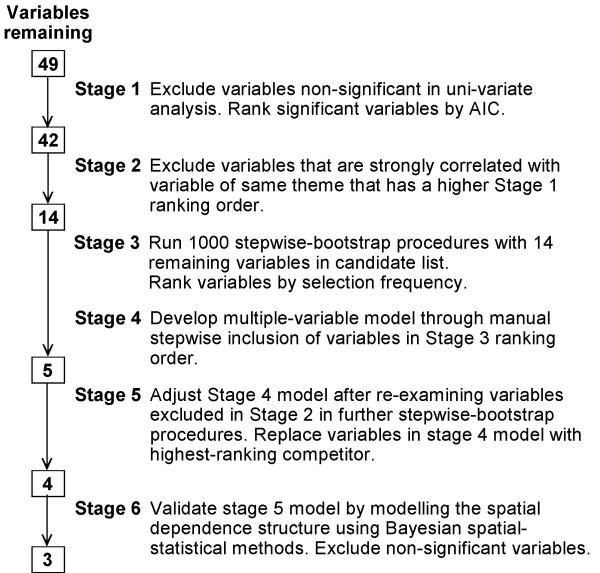
Flow diagram of staged variable selection procedure.

We carried out a staged approach during model formulation. A flow chart of the variable selection procedure is shown in figure 3.

#### Stage 1

The malaria prevalence database was split randomly into derivation (n = 81) and validation (n = 41) sub-sets. To identify the best univariate predictors, univariate logistic regression analysis against the derivation data was carried out on all 50 potential predictors. We allowed for clustering by survey location using the Hubert-White sandwich estimator in Stata [18].

#### Stage 2

**Figure 4:**
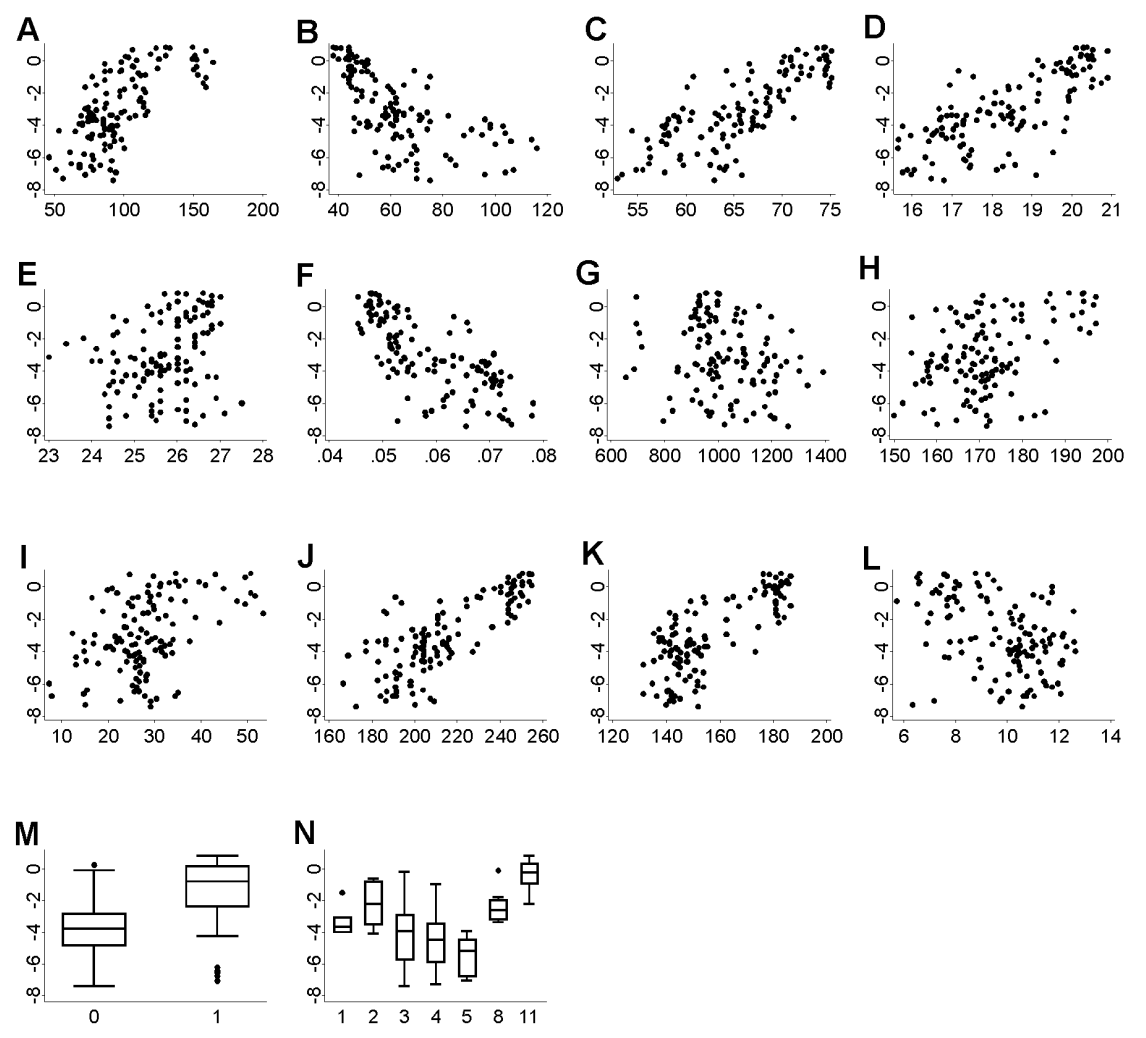
**Plots of malaria prevalence against fourteen potential explanatory variables**. Scatter – and box plots of candidate environmental explanatory variables used in step-wise procedures. Malaria prevalence in 1 to 14 year old children, Botswana, 1961/62, is shown on the Y axis on a logit scale. (A) annual maximum rainfall (mm); (B) winter (April – October) total rainfall (mm); (C) rainfall concentration (%); (D) winter (April – October) mean temperature (°C); (E) annual maximum temperature (°C); (F) temperature proportional standard deviation (°C); (G) elevation (m); (H) annual maximum NDVI; (I) NDVI standard deviation; (J) summer (December–March) mean vapour pressure (hPa); (K) vapour pressure standard deviation (hPa); (L) log distance to permanent water (m); (M) land cover: dry/low risk, moist/high risk areas; (N) start month of survey.

To reduce confounding arising from correlated variables, and also to reduce the variables to data ratio, we ranked the variables significant in univariate analysis by the Akaike Information Criterion [26] (AIC), and excluded those that were strongly correlated (Spearman's r > 0.85) with a higher-ranking variable belonging to the same environmental theme. Scatter plots against logit(p) were prepared of the remaining variables (figure 4).

#### Stage 3

Following the approach of Austin and Tu [11], we drew 1000 bootstrap samples from the derivation data, and ran automated backward exclusion procedures on each sample. Since it was not possible in Stata to allow for clustering within the stepwise procedure, which resulted in the explanatory power of variables being over-estimated, we used stringent entry and removal thresholds (p = 0.02 and 0.05 respectively). We recorded the co-efficients and the number of times each candidate variable was selected in the 1000 models.

#### Stage 4

A non-spatial multiple-variable model was derived in a manual step-wise fashion, starting with the most frequently selected variable, and adding further variables in order of selection frequency, as long as all entered variables remained significant at the 5% probability level. If a previously entered variable became non-significant with the addition of another, we retained the one more frequently selected in Stage 3 in favour of the other.

#### Stage 5

Back in Stage 2 variables had been excluded based on their univariate predictive power. To identify the best representative(s) of a theme in a multiple variable context, correlated variables excluded in Stage 2 were allowed to compete against each other for entry into the model in further stepwise-bootstrap procedures. The variables in the Stage 4 model constituted the basic candidate list. Working theme-by-theme, we re-introduced into the candidate list also those variables that had been excluded in Stage 2 on account of their high correlation with any variable of the same theme that had survived to Stage 4. Each time we ran a stepwise-bootstrap procedure as described above, recording which of the competitors was most frequently selected. This variable then replaced the original variable in the model. Details, in the form of an example, are provided in an annotation to table 2. Using the modified model, prevalence was predicted for all 122 observations. The accuracy of the predictions for both derivation and validation data was assessed using the concordance correlation coefficient [27,28].

**Table 2:** Results of bootstrap step-wise procedures. Variables included in the candidate lists of Stage 3 and Stage 5, and their selection frequency (fq), in four separate automated stepwise backward variable exclusion procedures, each time against 1000 bootstrap samples of the malaria prevalence data.

**Theme**	**Stage 3**		**Stage 5**					
	**Candidate variable list**	**fq**	**Candidate variable list 1‡**	**fq**	**Candidate variable list 2**	**fq**	**Candidate variable list 3**	**fq**
**Rainfall**								
	annual maximum *	904	annual maximum	560	annual maximum	533	annual maximum	914
			summer total †	821				
			number of months >80 mm	760				
			SD	726				
			total in months >80 mm	716				
			annual total	612				
	winter total	749						
	proportional SD	642						

**Temperature**								
	winter mean *	885	winter mean	993	winter mean	878	winter mean	665
					annual mean †	914		
					summer mean	885		
					number of months >16°C	681		
					mean in months >16°C	670		
					annual maximum	665		
					winter minimum	627		
					effective	615		
					annual minimum	558		
	proportional SD *	754	proportional SD	897	proportional SD	544	proportional SD	624
							SD	786
							annual range	537
	annual maximum	660						

**Vapour pressure**								
	SD	495						
	summer mean	441						

**NDVI**	annual maximum	567						
	SD	469						

Elevation *†		874	elevation	988	elevation	819	elevation	994
**Log distance to perennial water**		616						
Land cover *†		988	land cover	996	land cover	997	land cover	996
**Month of survey**		527						

NDVI – normalized difference vegetation index; SD – standard deviation

* Variables selected into Stage 4 model

† Variables selected into Stage 5 model

‡ Example: Five alternative rainfall indicators, listed in candidate list 1 under Stage 5, were strongly correlated with – and had been excluded in favour of – the annual maximum in Stage 2. In Stage 5, all six competing rainfall indicators were included in the candidate list, along with the other variables of the Stage 4 model. Of the six competitors the most frequently selected was summer total. In Stage 5 summer total therefore replaced annual maximum rainfall.

#### Stage 6

To account for spatial correlation in the survey data, a generalized geo-statistical spatial model using Markov Chain Monte Carlo (MCMC) simulation was fitted on all 122 observed prevalence data points [29-32]. The co-variates of the Stage 5 model were included as potential explanatory variables. Spatial modeling was carried out using the package geoRglm in the statistical software system R [30]. Detailed methods are included in the appendix. For each model parameter the median and 2.5 and 97.5 percentiles were calculated from the MCMC simulations. Prevalence and its 95% CI was predicted and mapped for a grid of 2300 locations based on the co-variates and the spatial structure in the data.

## Results

The design effect in the data was 52 before adjusting for co-variates. The clustered survey data thus only had the same power as 330 (17149/52) individuals randomly sampled over the entire country.

Of the 50 potential explanatory variables, 42 were significantly associated with malaria prevalence in univariate logistic regression in Stage 1 (table 1). Scatter plots of logit(p) against the 14 variables that were selected for further analysis in Stage 2, are shown in figure 4.

**Figure 5:**
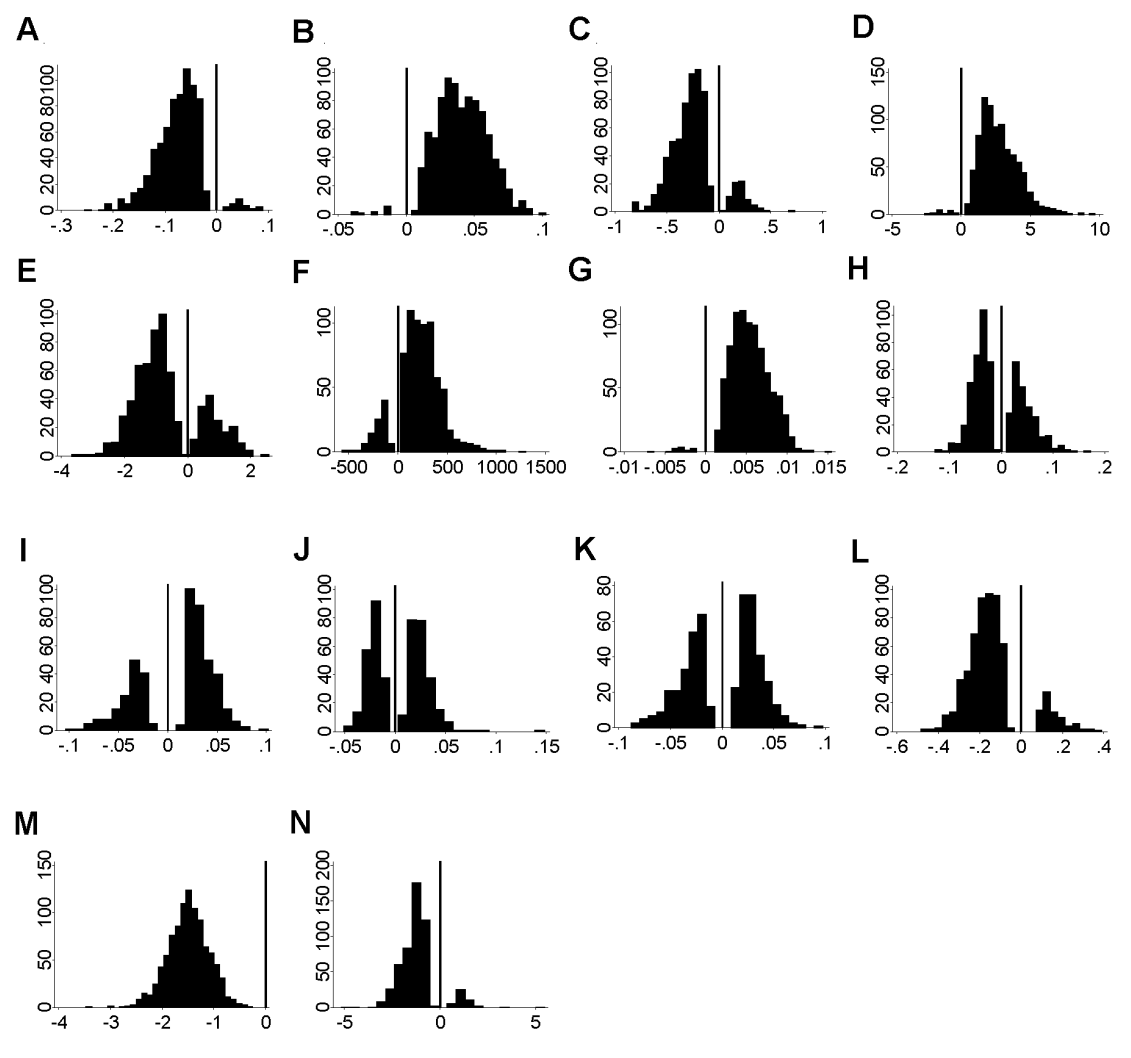
**Distribution of coefficients of fourteen candidate variables in 1000 stepwise bootstrap models**. Frequency histograms of coefficients obtained in automated backward stepwise exclusion regression analysis against 1000 bootstrap samples of the malaria prevalence data in Stage 3. In each case the vertical black line indicates coefficient = 0. (A) annual maximum rainfall (mm); (B) winter (April – October) total rainfall (mm); (C) rainfall concentration (%); (D) winter (April – October) mean temperature (°C); (E) annual maximum temperature (°C); (F) temperature proportional standard deviation (°C); (G) elevation (m); (H) annual maximum NDVI; (I) NDVI standard deviation; (J) summer (December–March) mean vapour pressure (hPa); (K) vapour pressure standard deviation (hPa); (L) log distance to permanent water (m); (M) land cover: dry/low risk, moist/high risk areas; (N) start month of survey: main season (April–May).

The selection frequency of the 14 candidate variables in the 1000 stepwise-bootstrap models of Stage 3, are shown in table 2. Figure 5 shows the frequency distribution of coefficients for each variable. Some variables were unstable, having positive coefficients in some models and negative coefficients in others. Five variables were selected into the Stage 4 model, namely annual maximum rainfall, winter mean temperature, proportional SD temperature, elevation and land cover (marked in table 2).

The results of the additional three stepwise-bootstrap procedures of Stage 5 are shown in table 2. In the rainfall theme, annual maximum was outperformed and replaced by summer total. For temperature theme, annual mean outperformed winter mean. With annual mean in the model, standard deviation became non-significant. Since standard deviation ranked lower in Stage 3 than winter mean, it was removed, reducing the number of variables in the Stage 5 model to four. Results of the Stage 5 model are shown in table 3.

**Table 3:** Results of the Stage 5 non-spatial model. Odds ratios, z-scores, and confidence interval estimated from non-spatial regression against four variables, fitted on derivation data only (n = 81, AIC = 8.06).

**Variable**	**Odds Ratio**	**z**	**p(z)**	**95%confidence interval**	
				**lower**	**upper**
rainfall summer total (per 100 mm)	2.33	6.9	<0.0005	1.84	2.99
temperature annual mean (per °C)	8.85	9.05	<0.0005	5.53	14.15
elevation (per 100 m)	1.68	3.8	<0.0005	1.28	2.20
high risk land cover	0.188	-5	<0.0005	0.098	0.361

**Figure 6:**
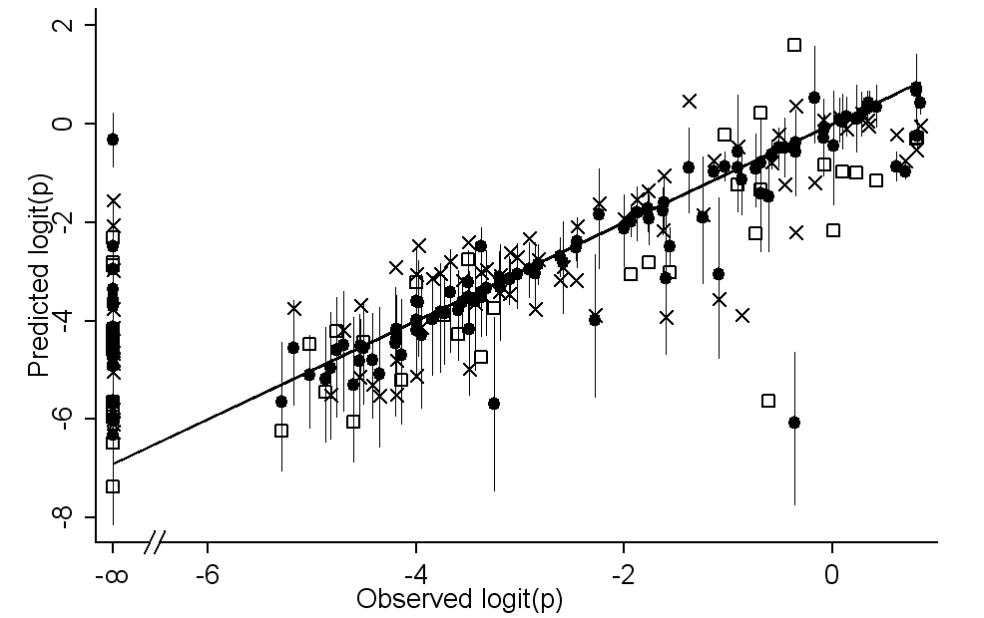
**Predicted *versus *observed prevalence**. Predicted *versus *observed prevalence, on a logit scale, for the derivation (crosses) and validation (squares) data of the Stage 5 non-spatial model, and for the median (closed circles) and upper/lower confidence interval (spikes) of the Stage 6 spatial model.

Figure 6 shows the scatter plot of observed *versus *predicted logit(p), for the derivation and validation data of the non-spatial Stage 5 model. The concordance correlation coefficient (*ρ*_C_) [27,28] for the derivation data, weighted by sample size, was 0.851, n (individuals examined) = 11182 in 66 non-zero prevalence surveys, the 95% confidence interval (CI) = 0.846 to 0.856. The unweighted *ρ*_C _= 0.834, n = 66, CI = 0.760 to 0.908. For the validation data weighted *ρ*_C _= 0.835, n = 4467, CI = 0.826 to 0.843; unweighted *ρ*_C _= 0.776, n = 30, CI = 0.635 to 0.917. The difference between observed and predicted logit(p) did not vary with prevalence.

After adjusting for spatial random effects, only three co-variates remained significant. Land cover (median = -0.515; 95% CI = -1.099 and 0.059) was removed. The predictions (median and CI) from the spatial Stage 6 model are also shown in figure 6. It contained three co-variates namely summer rainfall, annual mean temperature and elevation, each independently significantly associated with prevalence of infection after allowing for spatial correlation in the data (table 4).

**Table 4:** Results of the Stage 6 spatial model. Odds ratios and confidence interval estimated from Stage 6 spatial model, fitted on all prevalence data (n = 122).

**Variable**	**Odds Ratio**	**95%confidence interval**	
		**lower**	**upper**
rainfall summer total (per 100 mm)	2.01	1.49	2.70
temperature annual mean (per °C)	5.75	4.14	8.08
elevation (per 100 m)	1.82	1.49	2.22

Φ = 0.003, 95% CI = 0, 0.0174, *σ*^2 ^= 0.77, 95% credible interval (0.53, 1.14)

## Discussion

This study was concerned with finding the best predictors of malaria prevalence in terms of plausibility, parsimony and reliability. One important question was how to summarize the environmental data in a meaningful way. We determined to explore a range of alternative summaries of the monthly climate data, believing one appropriate summary indicator to be better for prediction than individual months [5], quarterly aggregates [3], or principal components [4], the last of which are difficult to interpret. However, as more and more variables are tested against a certain data set, the risk increases that some will explain the data merely by chance, but will fail to explain new data.

In an initial attempt to derive a well-fitting and plausible model through automated step-wise variable selection (results not shown), arbitrary factors such as entry and removal threshold settings, how many variables were included in the list of candidates, and which data-subset was used for model derivation, affected which variables got selected. The best-fitting models did not produce the most plausible risk maps, and *visa versa*. The majority of maps resulting from these models strongly contradicted expert opinion. A more systematic selection procedure was called for.

Identification of consistent predictors is compromised by correlation among predictors. A strong, reliable predictor may ultimately be selected less frequently than a weaker predictor, if several strongly correlated alternatives compete for entry into the model so that each has a low selection frequency [11]. For this reason it was important to include in the candidate list only little-correlated variables. This was ensured in Stage 2, where the candidate list was reduced from 42 to 14.

Reliable predictors would not only explain a particular data set, but would be associated consistently with the response. The bootstrapping of Stage 3 helped identify such predictors, because those that consistently explain different sub-sets of the data, are more likely to explain new data. In the step-wise bootstrap procedures, variables that explained the most observations would be selected most frequently while those that explained only some of the observations, would be selected only when these observations appeared in the bootstrap sample. The effect of individual observations on variable selection, especially of outliers, was thus reduced.

In the process of uni-variate ranking (Stage 1 and 2) we became guilty of "data peeking" [9]. Using our data to assemble a candidate list of predictors set up the analysis for success. Such undeclared testing and discarding of variables may lead to illegitimately high model fit. Another problem of Stage 2 was that variables were excluded on the grounds of low uni-variate correlation with the response, while their predictive power may be quite different once other variables are accounted for. Stage 5 was an attempt to redress both these problems at once, by giving each variable excluded in Stage 2, whose relative had survived up to Stage 4, a fair chance to out-perform and supplant its competitors in a multiple-variable context, at the same time, through the bootstrap sub-sampling, to reduce the influence of the data set on this process.

A further benefit of the Stage 3 bootstrap-stepwise procedures was the information provided by the frequency distributions of coefficients in the 1000 stepwise models (figure 5). A variable that has a widely varying coefficient, or one that is sometimes positive and sometimes negative, is clearly not reliable and should be considered with suspicion [33]. An example was summer vapour pressure, the strongest uni-variate predictor, but selected least frequently in multiple-variable regression (figure 5J ). Altitude on the other hand, a weak uni-variate predictor, became an important predictor in a multiple-variable context, with a stable positive coefficient (figure 5G ). In fact, the most frequently selected variables (table 2) had stable coefficients (figure 5), whereas the most unstable coefficients were found among the least frequently selected variables, confirming the relative importance of predictors.

**Figure 7:**
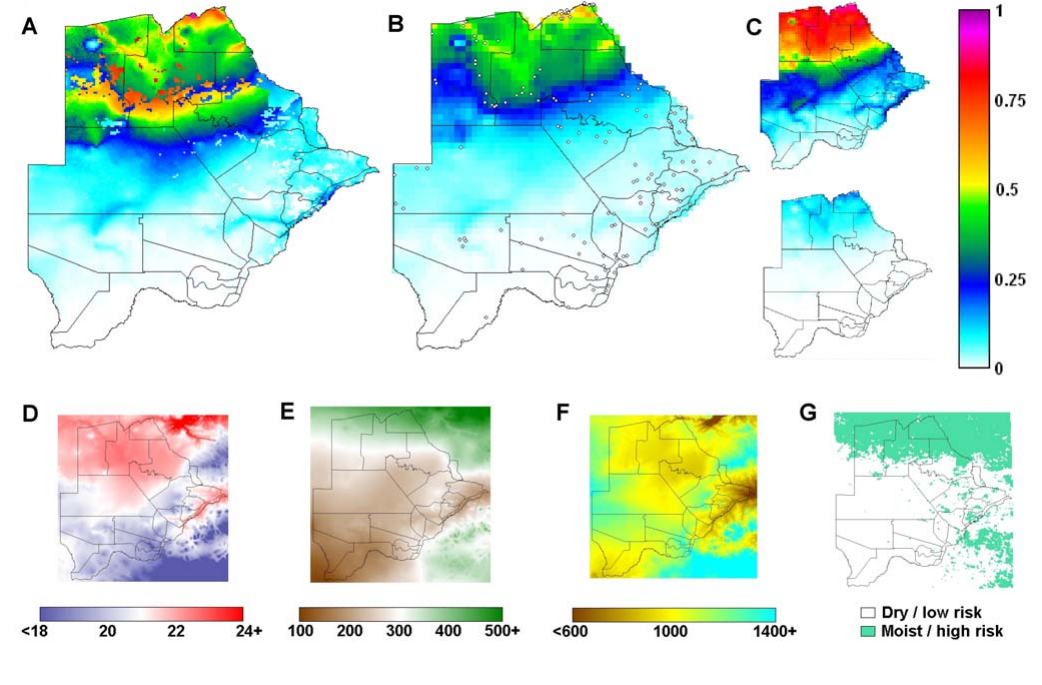
**Maps of predicted malaria prevalence and covariates**. Predicted pre-control childhood malaria prevalence maps for Botswana, resulting from (A) the stage 5 non-spatial model and (B) the stage 6 spatial model; 118 survey sites are shown; (C) the upper and lower 95% CI of the spatial model. Co-variates used in the models: (D) annual mean temperature, C; (E) summer total rainfall, mm; (F) elevation, m; (G) land cover categories, high-risk/low-risk. Lines represent district boundaries.

The strong association found between malaria prevalence and selected environmental data (figure 7) is biologically plausible since high malaria infections have been shown to coincide with conditions that favour vector and parasite development in a given location [3]. However, over small distances environmental conditions vary only slightly due to the relatively simple flat Botswanan topography, while malaria prevalence showed substantial local variation, for example contemporal measures of 67% (n = 48, Maun) *versus *24% (n = 557, Maun suburb), or 3% (n = 219) *versus *17% (n = 116) in Matangwane. Such local variation is perhaps partly caused by the distribution of small breeding sites. Yet in studies where detailed breeding site information was available, much of the variation in incidence [34], prevalence and entomologic inoculation rate [35] nevertheless remained unexplained. Localized factors, such as individual, household and village characteristics, as well as the effect of sampling procedure and size, may further contribute to the unexplained variability in prevalence.

Summer rainfall and annual mean temperature, retained in the final multiple-variable model, were highly plausible predictors. The same variables – summer rain and mean temperature over the preceding year – were also found to explain inter-seasonal variation in malaria incidence in KwaZulu-Natal [36]. Summer rainfall also explained much of the variation in inter-annual variation in malaria incidence in Botswana [16]. High rainfall during the hot summer months allows rapid breeding and population expansion of the mosquito vectors, while high mean temperatures maximize the maturation rate of the parasite in its exothermic arthropod host [37]. Warmer winters reduce the die-back of mosquitoes and parasites, thereby increasing the reservoir for the following season.

The strong positive association of elevation with malaria prevalence (an increase in logit(p) of 1 every 160 m, table 4) was surprising, as prevalence on its own, as it usually tends to be, was higher in low-lying areas (figure 4G). This positive association was difficult to explain, but may be connected with the malaria control that was ongoing at the time. It appears from early reports [15] that vector control operations were wide-spread and intensive along rivers and the main populated areas.

The non-spatial model of Stage 5 predicted the data fairly well but the predictions achieved by the spatial model of Stage 6 were more accurate (figure 6). The map corresponding to the Stage 5 model (figure 7A) had an implausible discontinuity, caused by the negative co-efficient of land-cover. Land-cover was the most frequently selected variable in the bootstrap procedures, but was not significant in the spatial model. This binary variable may simply have approximated the spatial division between high and low prevalence areas, which was ultimately described more correctly through the geo-spatial approach of Stage 6 (figure 7B).

A good number of locations with observed zero prevalence had predicted prevalence of 5%, i.e. logit(p) of -3, and above (figure 6). In these cases sampling error may have played an important role, as large sample sizes are needed to measure very low prevalence rates confidently. Conversely, non-zero observations were more often lower than the predictions based on environmental factors. By 1961/2 malaria prevalence in the North of Botswana was already much below the level measured in 1944 [14], probably due to the limited use of indoor residual spraying which had been ongoing since the 1940's. This highlights the fact that not only environmental, but also anthropogenic factors, especially malaria control need to be considered. This furthermore highlights the need to monitor control coverage and effectiveness, as well as other potential cofactors, in order to understand the situation more accurately.

Evidence from elsewhere in Africa suggests that prevalence rates in the dry/low transmission season may differ substantially from those in the wet/high transmission season [38,39]. In this study month of survey was a significant predictor of prevalence in a univariate setting only, but not while accounting for other variables. Prevalence by month (figure 4N) was confounded by where surveys were carried out when, and thus did not reflect the seasonality of malaria risk. The highest incidence months for example (March to May) would not be the lowest prevalence months, as figure 4N suggests. Rather, surveys were carried out during these months in the low-risk South (figure 2). To measure intra-annual variation in prevalence we would have required data from the same localities in different months.

The spatial risk map (figure 7B) presents a smoothed picture of malaria risk in Botswana prior to intensive malaria control, which was highly plausible based on expert opinion and the mean incidence at district level [40]. The wide CI (figure 7C) in predicted prevalence highlights the uncertainty remaining after accounting for all explained variation in the data. The confidence level needs to be taken into account when using the map for planning and evaluating control interventions, to avoid over-interpretation of the map.

## Conclusion

A continuous map of malaria risk is more useful than point-prevalence rates for several reasons. First, the variability in individual observations may hide underlying patterns that have epidemiological importance. Further, it is not possible to deduce from a point-referenced map what prevalence you may expect to see in areas that have not been sampled, whereas a model such as the one developed here gives a likely range of prevalence for the entire region. A continuous prevalence map can also be combined with underlying population data to estimate the number of people at risk of – or infected with – malaria. Finally, the spatial statistical methods employed here distinguish between the correlation among observations that can be ascribed to their spatial proximity (neighbouring villages affecting each other), and that which can be explained by environmental factors (thereby avoiding overestimating the explanatory power of the covariates).

Though malaria risk has been reduced substantially through intense malaria control, a malaria risk map nevertheless remains highly useful from the control perspective in knowing historical prevalence levels. We have furthermore demonstrated a systematic procedure for variable selection and model formulation in developing a geo-statistical risk model from point-referenced malaria prevalence data, which has relevance to a broad range of environmentally determined infectious diseases. The failure take account of spatial correlations during the entire variable selection procedure remained a major weakness. As computing power increases and statistical software packages are further developed, variable selection within a spatial framework may end up being within the means of the average researcher.

The staged process of variable elimination employed here proved to be practical, though not necessarily the optimal solution. Stepwise variable selection on multiple bootstrap samples drawn from the data allowed us to identify the most consistent and stable explanatory variables. Selection frequency provided an objective rationale for choosing one variable above another, and to choose between similar and strongly correlated indicators. Spatial analysis was the final stage in the variable elimination process, after which we remained with a parsimonious, highly plausible model, which produced a smooth, plausible map of malaria risk.

## Appendix 1

### Standard deviation (SD)

SD=∑m=112(y^−ym)2
 MathType@MTEF@5@5@+=feaafiart1ev1aaatCvAUfKttLearuWrP9MDH5MBPbIqV92AaeXatLxBI9gBaebbnrfifHhDYfgasaacH8akY=wiFfYdH8Gipec8Eeeu0xXdbba9frFj0=OqFfea0dXdd9vqai=hGuQ8kuc9pgc9s8qqaq=dirpe0xb9q8qiLsFr0=vr0=vr0dc8meaabaqaciaacaGaaeqabaqabeGadaaakeaacqqGtbWucqqGebarcqGH9aqpdaGcaaqaamaaqahabaGaeiikaGIafmyEaKNbaKaacqGHsislcqWG5bqEdaWgaaWcbaGaemyBa0gabeaakiabcMcaPmaaCaaaleqabaGaeGOmaidaaaqaaiabd2gaTjabg2da9iabigdaXaqaaiabigdaXiabikdaYaqdcqGHris5aaWcbeaaaaa@3FD8@

where y_m _= monthly value and y^
 MathType@MTEF@5@5@+=feaafiart1ev1aaatCvAUfKttLearuWrP9MDH5MBPbIqV92AaeXatLxBI9gBaebbnrfifHhDYfgasaacH8akY=wiFfYdH8Gipec8Eeeu0xXdbba9frFj0=OqFfea0dXdd9vqai=hGuQ8kuc9pgc9s8qqaq=dirpe0xb9q8qiLsFr0=vr0=vr0dc8meaabaqaciaacaGaaeqabaqabeGadaaakeaaieaacuWF5bqEgaqcaaaa@2E3C@ = mean of all y_m_.

### Proportional SD (based on monthly proportions)

Proportional SD=∑m=112(0.0833−pym)2
 MathType@MTEF@5@5@+=feaafiart1ev1aaatCvAUfKttLearuWrP9MDH5MBPbIqV92AaeXatLxBI9gBaebbnrfifHhDYfgasaacH8akY=wiFfYdH8Gipec8Eeeu0xXdbba9frFj0=OqFfea0dXdd9vqai=hGuQ8kuc9pgc9s8qqaq=dirpe0xb9q8qiLsFr0=vr0=vr0dc8meaabaqaciaacaGaaeqabaqabeGadaaakeaacqqGqbaucqqGYbGCcqqGVbWBcqqGWbaCcqqGVbWBcqqGYbGCcqqG0baDcqqGPbqAcqqGVbWBcqqGUbGBcqqGHbqycqqGSbaBcqqGGaaicqqGtbWucqqGebarcqGH9aqpdaGcaaqaamaaqahabaGaeiikaGIaeGimaaJaeiOla4IaeGimaaJaeGioaGJaeG4mamJaeG4mamJaeyOeI0IaemiCaaNaemyEaK3aaSbaaSqaaiabd2gaTbqabaGccqGGPaqkdaahaaWcbeqaaiabikdaYaaaaeaacqWGTbqBcqGH9aqpcqaIXaqmaeaacqaIXaqmcqaIYaGma0GaeyyeIuoaaSqabaaaaa@5689@

where py_m _= y_m_/y_tot_; y_tot _= ∑y_m_, and 0.0833 is the mean of all py_m _(= 1/12)

### Effective temperature [41]

Effective temperature = [8 * annual mean + 14 * annual range]/[8 + annual range]

### Concentration of rainfall

Monthly rainfall is expressed as a vector (*r*_*m*_, *θ*_*m*_), rainfall being the magnitude (*r*) of the vector and the month its angle (*θ*) expressed in units of arc:

*θ*_*m *_= *m*2*π*/12

where *m *is the month, so that January = 1 and December = 12.

The twelve monthly vectors are added to calculate the total vector (*r*_*t*_, *θ*_*t*_):

rt=(∑m=112rmcos⁡θm)2+(∑m=112rmsin⁡θm)2
 MathType@MTEF@5@5@+=feaafiart1ev1aaatCvAUfKttLearuWrP9MDH5MBPbIqV92AaeXatLxBI9gBaebbnrfifHhDYfgasaacH8akY=wiFfYdH8Gipec8Eeeu0xXdbba9frFj0=OqFfea0dXdd9vqai=hGuQ8kuc9pgc9s8qqaq=dirpe0xb9q8qiLsFr0=vr0=vr0dc8meaabaqaciaacaGaaeqabaqabeGadaaakeaacqWGYbGCdaWgaaWcbaGaemiDaqhabeaakiabg2da9maakaaabaWaaeWaaeaadaaeWbqaaiabdkhaYnaaBaaaleaacqWGTbqBaeqaaOGagi4yamMaei4Ba8Maei4CamhcciGae8hUde3aaSbaaSqaaiabd2gaTbqabaaabaGaemyBa0Maeyypa0JaeGymaedabaGaeGymaeJaeGOmaidaniabggHiLdaakiaawIcacaGLPaaadaahaaWcbeqaaiabikdaYaaakiabgUcaRmaabmaabaWaaabCaeaacqWGYbGCdaWgaaWcbaGaemyBa0gabeaakiGbcohaZjabcMgaPjabc6gaUjab=H7aXnaaBaaaleaacqWGTbqBaeqaaaqaaiabd2gaTjabg2da9iabigdaXaqaaiabigdaXiabikdaYaqdcqGHris5aaGccaGLOaGaayzkaaWaaWbaaSqabeaacqaIYaGmaaaabeaaaaa@5AF4@

θt=tan⁡−1(∑m=112rmsin⁡θm∑m=112rmcos⁡θm)
 MathType@MTEF@5@5@+=feaafiart1ev1aaatCvAUfKttLearuWrP9MDH5MBPbIqV92AaeXatLxBI9gBaebbnrfifHhDYfgasaacH8akY=wiFfYdH8Gipec8Eeeu0xXdbba9frFj0=OqFfea0dXdd9vqai=hGuQ8kuc9pgc9s8qqaq=dirpe0xb9q8qiLsFr0=vr0=vr0dc8meaabaqaciaacaGaaeqabaqabeGadaaakeaaiiGacqWF4oqCdaWgaaWcbaGaemiDaqhabeaakiabg2da9iGbcsha0jabcggaHjabc6gaUnaaCaaaleqabaGaeyOeI0IaeGymaedaaOWaaeWaaeaadaWcaaqaamaaqahabaGaemOCai3aaSbaaSqaaiabd2gaTbqabaGccyGGZbWCcqGGPbqAcqGGUbGBcqWF4oqCdaWgaaWcbaGaemyBa0gabeaaaeaacqWGTbqBcqGH9aqpcqaIXaqmaeaacqaIXaqmcqaIYaGma0GaeyyeIuoaaOqaamaaqahabaGaemOCai3aaSbaaSqaaiabd2gaTbqabaGccyGGJbWycqGGVbWBcqGGZbWCcqWF4oqCdaWgaaWcbaGaemyBa0gabeaaaeaacqWGTbqBcqGH9aqpcqaIXaqmaeaacqaIXaqmcqaIYaGma0GaeyyeIuoaaaaakiaawIcacaGLPaaaaaa@5CB9@

The concentration index C is calculated as:

C = 100*r*_*t*_/annual total

Concentration is 100% if all the rain falls in one month and 0% if all months have equal amount of rain.

*θ*_*t *_is the mean peak month around which rainfall is concentrated.

### Generalized spatial logistic regression analysis

Bayesian geostatistical model formulation has been described by a number of authors [29-32]. Following these authors, the model is specified as follows:

Y_ji _represents the binary response corresponding to the infection status of child *j *at site *i *(the survey site) taking value 1 if the child tested positive and 0 otherwise. The Y_ji _are conditionally independent Bernoulli variables with infection probability p_i _at location *i*.

The p_i _are defined via a generalised linear mixed model, to take account of spatial dependence:

logit(p_i_) = **X**_i_**β**+S(ℓ_i_)

where **β **represents the regression coefficients for a set of known covariates **X **at all locations ℓ_i _of the study area;

S = (S(ℓ_1_),...., S(ℓ_n_))^T ^denotes the values of the (unobserved) Gaussian spatial process S(·) at sample locations ℓ_i_;

*σ*^2 ^= Var{S(ℓ)}, and Φ is a parameter of the correlation function *ρ*(d_ij_, Φ), in our case exp(-d_ij_/Φ), where d_ij _is the distance between locations ℓ_*i *_and ℓ_*j*_.

For **β **flat priors were specified respectively (defaults in geoRglm) and for *σ*^2 ^a Scaled-Inverse chisquare distribution(*χ*^2^_ScI_) with five degrees of freedom and a mean of 0.5. For Φ a discrete exponential prior with mean of 0.04 and 1000 discretisation points in the interval 0.0001 to 2 was specified.

Convergence was assessed by inspecting plots of traces of simulations for individual parameters. The first 50,000 iterations were discarded; thereafter simulations were run for 250,000 iterations. Every 50th sample was retained. For each model parameter the median and 2.5 and 97.5 percentiles were calculated from the 5,000 MCMC simulations.

Models were compared by calculating the deviance information criterion (DIC) for each model [42]. Spatial prediction using Bayesian kriging was carried out for a grid of 2300 locations which correspond to the entire surface of Botswana. For each prediction location a posterior sample of MCMC simulations was generated taking account of the estimates of regression coefficients and the spatial effects at each location, and of the uncertainty of each parameter. This process is described in detail elsewhere [29,31,32], and was carried out using geoR [30].

## Abbreviations

CI Confidence interval/credible interval

logit(p) Logit-transformed malaria prevalence

MCMC Markov Chain Monte Carlo

NDVI Normalized difference vegetation index

SD Standard deviation

## Competing interests

The author(s) declare that they have no competing interests.

## Authors' contributions

All authors critically reviewed several versions of the manuscript. MC carried out the bulk of the analysis and drafted the manuscript. IK participated in its design, carried out the spatial analysis and helped draft the manuscript. MM collated the malaria data. MC, IK and MM read and approved the final manuscript. BS recently passed away and we hereby wish to express our deepest appreciation of his leadership, his personal and professional support over the years and his tireless efforts towards malaria control.

## References

[B1] Snow RW, Marsh K, Le Sueur D (1996). The need for maps of transmission intensity to guide malaria control in Africa. Parasitol Today.

[B2] Kleinschmidt I, Bagayoko M, Clarke GP, Craig M, Le Sueur D (2000). A spatial statistical approach to malaria mapping. Int J Epidemiol.

[B3] Kleinschmidt I, Omumbo J, Briet O, Van De GN, Sogoba N, Mensah NK, Windmeijer P, Moussa M, Teuscher T (2001). An empirical malaria distribution map for West Africa. Trop Med Int Health.

[B4] Omumbo JA, Hay SI, Snow RW, Tatem AJ, Rogers DJ (2005). Modelling malaria risk in East Africa at high-spatial resolution. Trop Med Int Health.

[B5] Snow RW, Gouws E, Omumbo JA, Rapuoda B, Craig MH, Tanser FC, Le Sueur D, Ouma J (1998). Models to predict the intensity of *Plasmodium falciparum *transmission: applications to the burden of disease in Kenya. Trans R Soc Trop Med Hyg.

[B6] Gemperli A, Vounatsou P, Sogoba N, Smith T (2006). Malaria mapping using transmission models: application to survey data from Mali. Am J Epidemiol.

[B7] Justice AC, Covinsky KE, Berlin JA (1999). Assessing the generalizability of prognostic information. Ann Intern Med.

[B8] Diggle P, Moyeed R, Rowlingson B, Thomson M (2002). Childhood malaria in the Gambia: a case-study in model-based statistics. Applied Statistics.

[B9] Babyak MA (2004). What you see may not be what you get: a brief, nontechnical introduction to overfitting in regression-type models. Psychosom Med.

[B10] Harrell FE (2001). Regression modeling strategies: with applications to linear models, logistic regression and survival analysis.

[B11] Austin PC, Tu JV (2004). Bootstrap methods for developing predictive models. Am Stat.

[B12] Deichmann U (1997). Population Density for Africa in 1990, 3. Internet.

[B13] Chayabejara S, Sobti SK, Payne D, Braga F Malaria situation in Botswana. Report AFR/MAL/144.

[B14] Mabaso ML, Sharp B, Lengeler C (2004). Historical review of malarial control in southern African with emphasis on the use of indoor residual house-spraying. Trop Med Int Health.

[B15] Freedman ML Malaria Control. Report.

[B16] Thomson MC, Mason SJ, Phindela T, Connor SJ (2005). Use of rainfall and sea surface temperature monitoring for malaria early warning in Botswana. Am J Trop Med Hyg.

[B17] Omumbo J, Ouma J, Rapuoda B, Craig MH, Le Sueur D, Snow RW (1998). Mapping malaria transmission intensity using geographical information systems (GIS): an example from Kenya. Ann Trop Med Parasitol.

[B18] Anon (2001). Stata Statistical Software: Release 7.0.

[B19] Anon (1998). GTOPO30 global digital elevation model. Internet.

[B20] Anon (1995). Africa Data Sampler, 1. CD-ROM.

[B21] Anderson JR, Hardy EE, Roach JT, Witmer RE (1976). A land use and land cover classification system for use with remote sensor data. U S Geological Survey Professional Paper.

[B22] Hutchinson MF, Nix HA, McMahan JP, Ord KD (1995). Africa – A topographic and climatic database, 1. CD-ROM, Canberra.

[B23] Mitchell TD, Hulme M, New M (2003). CRU TS 2.0 high-resolution gridded climate data, 1. Internet.

[B24] Anon (2001). Pathfinder Advanced Very High Resolution Radiometer (AVHRR) data. Internet.

[B25] Anon (2007). Global Land 1-KM AVHRR Project. Internet.

[B26] Akaike H, Petrov BN, Csaki F (1973). Information theory and an extension of the maximum likelihood principle. Second international symposium on information theory.

[B27] Lin LIK (1989). A concordance correlation coefficient to evaluate reproducibility. Biometrics.

[B28] Lin LIK (2000). A note on the concordance correlation coefficient. Biometrics.

[B29] Diggle PJ, Tawn JA, Moyeed R (1998). Model-based geostatistics. J Roy Stat Soc C.

[B30] Christensen OF, Ribeiro PJJ (2002). geoRglm – a package for generalised linear spatial models. R News.

[B31] Gemperli A, Vounatsou P (2003). Fitting generalized linear mixed models for point-referenced spatial data. J Modern Appl Stat Meth.

[B32] Gemperli A, Vounatsou P, Kleinschmidt I, Bagayoko M, Lengeler C, Smith T (2004). Spatial patterns of infant mortality in Mali: the effect of malaria endemicity. Am J Epidemiol.

[B33] Concato J, Feinstein AR, Holford TR (1993). The risk of determining risk with multivariable models. Ann Intern Med.

[B34] Van Der Hoek W, Konradsen F, Amerasinghe PH, Perera D, Piyaratne M, Amerasinghe FP (2003). Towards a risk map of malaria for Sri Lanka: the importance of house location relative to vector breeding sites. Int J Epidemiol.

[B35] Hightower AW, Ombok M, Otieno R, Odhiambo R, Oloo AJ, Lal AA, Nahlen BL, Hawley WA (1998). A geographic information system applied to a malaria field study in western Kenya. Am J Trop Med Hyg.

[B36] Craig MH, Kleinschmidt I, Nawn JB, Le Sueur D, Sharp BL (2004). Exploring 30 years of malaria case data in KwaZulu-Natal, South Africa, Part I: the impact of climatic factors. Trop Med Int Health.

[B37] Molineaux L, Wernsdorfer WH, McGregor I (1988). The epidemiology of human malaria as an explanation of its distribution, including some implications for its control. Malaria: Principles and Practice of Malariology.

[B38] Lindsay SW, Wilkins HA, Zieler HA, Daly RJ, Petrarca V, Byass P (1991). Ability of Anopheles gambiae mosquitoes to transmit malaria during the dry and wet seasons in an area of irrigated rice cultivation in The Gambia. J Trop Med Hyg.

[B39] Molineaux L, Gramiccia G (1980). The Garki project: research on the epidemiology and control of malaria in the Sudan savanna of West Africa.

[B40] Craig MH, Snow RW, Le Sueur D (1999). A climate-based distribution model of malaria transmission in Africa. Parasitol Today.

[B41] Stuckenberg BR (1969). Effective temperature as an ecological factor in southern Africa. Zool Afr.

[B42] Spiegelhalter DJ, Best NG, Carlin BP, Linde AVD (2002). Bayesian measures of model complexity and fit. J Roy Stat Soc B.

